# Design and Development of Novel Dual-Compartment Capsule for Improved Gastroretention

**DOI:** 10.1155/2013/752471

**Published:** 2013-01-27

**Authors:** Ganesh B. Patil, Saurabh S. Singh, Ketan P. Ramani, Vivekanand K. Chatap, Prashant K. Deshmukh

**Affiliations:** Post-Graduate Department of Pharmaceutics, H R Patel Institute of Pharmaceutical Education and Research, Karvand Naka, Dhule District, Shirpur, Maharashtra 425 405, India

## Abstract

The aim of the proposed research work was to develop a novel dual-compartment capsule (NDCC) with polymeric disc for gastroretentive dosage form, which will ultimately result in better solubility and bioavailability of Ofloxacin. Floating ring caps were formulated by using different natural polymers, separating ring band and swellable polymer located at the bottom of capsule. Formulated ring caps were assessed for coating thickness, *In vitro* buoyancy, *In vitro* drug release, release kinetics and stability studies. Coating attained by the capsule shell was found to be 0.0643 mm. Depending on nature of natural polymer used, most of the formulations showed buoyancy for more than 9 hrs. Developed formulation demonstrated considerably higher drug release up to 9 hrs. The developed formulation F_E2_ depicted the drug release according to Korsmeyer-Peppas model. There was not any significant change in performance characteristics of developed ring caps after subjecting them to stability studies. The present study suggests that the use of NDCC for oral delivery of Ofloxacin could be an alternative to improve its systemic availability which could be regulated by the floating approach. The designed dosage system can have futuristic applications over payloads which require stomach-specific delivery.

## 1. Introduction

Although tremendous advances have been made in drug delivery, considering costs and patient compliance, the oral route still remains the preferred route of administration for therapeutic agents. The environment of gastrointestinal tract significantly varies from stomach to large intestine (Table 1) [1]. This variation could serve a promising platform for the site-specific drug delivery of therapeutics.

The presence of a dosage form in the upper part of the gastrointestinal tract is important especially for drugs that are degraded or metabolized in the intestine or for drugs with local activity in the stomach [2, 3]. Likewise Singh and Kim [4] suggested that floating drug delivery is of particular interest for drugs which (a) have local action in the stomach, (b) are primarily absorbed in the stomach, (c) have poor solubility at an alkaline pH, (d) have a narrow window of absorption, and (e) are unstable in the intestinal or colonic environment. Gastrointestinal retention depends on many factors such as density and size of the dosage form, the fasting or fed condition of the patient, and the nature of the meal as well as posture [5–7]. Several gastroretentive formulation approaches such as high density [8], swelling [9], bioadhesive [10], magnetic [11], and floating [12] systems have been developed for enhanced gastroretention.

Local action in stomach is often used for curing gastric infection and better bioavailability of drugs which shows pH-dependent solubility. Ofloxacin is known to have pH-dependant solubility; it is more soluble in acidic pH and slightly soluble at neutral or alkaline pH conditions [13].

In the present investigation, for better solubility and bioavailability of ofloxacin an attempt was made to develop (gastroretentive drug delivery system) (GRDS) of ofloxacin by fabricating it in form of novel dual-compartment capsule (NDCC) for gastroretentive dosage form. The present study also reveals the effects of different polymers with varying concentration on drug release and floating property of prepared formulation.

The objective of the current investigation was to develop a novel dual-compartment capsule (NDCC) with polymeric disc for gastroretentive dosage form. The study was inspired from patented RingCap technology for enhanced drug release by augmentation of surface area approach and OROS push-pull osmotic system. 

## 2. Materials and Methods

### 2.1. Materials

Ofloxacin was generously gifted by Ajanta Pharma, Mumbai, India. Carbopol 934P, Carbopol 940P, HPMC A15, HPMC E15, and HPMC K15 were procured from HI-MEDIA, Eudragit S100 was purchased from Loba Chemie, Mumbai, and Xanthan gum, Guar gum, Sodium Alginate, and Glycerol were procured from Merck Mumbai, India. All other chemicals and reagents used were of analytical grade.

### 2.2. Solubility of Ofloxacin in Different pH Conditions

In concordance with Chavanpatil et al. the study was conducted to depict the solubility of ofloxacin at varying pH conditions. Saturated solutions of ofloxacin were prepared by dispersing an excess amount of drug in the buffer with pH 1, 1.2, 3, 4.5, 5.8, 6.8, 7.2, and 7.5. The samples were subjected to orbital shaker (Remi Instrument Ltd. CIS-24) for 72 hours to aid maximum dissolution of the drug. After the incubation period, clear saturated solutions were obtained by filtration (0.45 *μ*m Millipore filter paper), and the concentration of the drug was determined spectrophotometrically (UV-1800 PC Shimadzu, Japan) at the wavelength of 291 nm, after the appropriate dilution in the corresponding pH buffer. All solubilities were measured in triplicate at room temperature [14].

### 2.3. Preparation of Separating Polymeric Disc

It was prepared by making 4% HMPC K15 solution in water, followed by the addition of 5% glycerol which was preoptimized. Then the mixture was poured to petridish and was allowed to dry at 45°C for 12 hours. The dried film was cut uniformly to obtain circular discs with punch to have 1 mm thickness and diameter of 5.1 mm.

### 2.4. Preparation of Enteric Coated Gelatin Body

Enteric coated gelatin bodies were coated in a coating pan rotated at 50 rpm by spraying with a 10% solution of Eudragit S100 in acetone to get uniform coating over the surface of gelatin body. As Eudragit S100 is insoluble in gastric environment and buffer solutions with pH below 6, thus it provides protection to the prepared formulation [15, 16].

### 2.5. Fabrication of NDCC

In enteric coated gelatin body, initially 50 mg Carbopol 934P was placed at the base; over this circular separating polymeric disc of 1 mm thickness it was placed.

As shown in Table 2, prepared polymeric mixture of ofloxacin in combination with different polymers was placed over this barrier disc. Thereafter uncoated gelatin cap was joined over the enteric body of capsule to complete the design of NDCC. The polymers used with model drug are swellable and show hydration when they come in contact with acidic media of stomach. As the enteric gelatin body remains intact in acidic environment, it protects the formulation and allows the exposure only from open face. The disc pushes the drug polymeric mixture due to the swelling of Carbopol 934P on hydration which is located deep at the base. When the prepared capsule was assessed *in vitro* in simulated gastric fluid, it was observed that the cap quickly gets dissolved, but the enteric body still remains intact. Air trapped inside the intact capsule shell offers the buoyancy.

### 2.6. Evaluation of Coating Thickness

The prepared enteric coated capsules were evaluated for the thickness attained by Eudragit S100 coating. The thickness was measured by Digimatic caliper (Mitutoyo Corporation, Japan). Initially, the bare capsule shells were subjected for thickness measurement at three different positions. This was followed by final measurement at three different positions of the coated capsules. The changes in thickness indicate the final Eudragit coating acquired by the capsules. The evaluation was done in triplicate:

(1) Coating  thickness=Thickness  of  coated  capsule−Thickness  of  bare  capsule.

### 2.7. Preparation of Novel Floating Capsules

**Figure 1:**
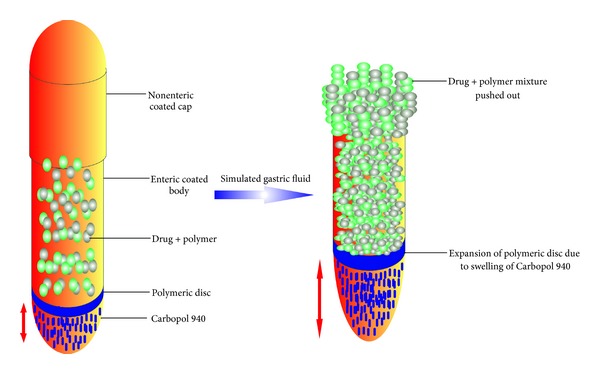
Schematic representation of drug release from novel dual compartment capsule.

Enteric gelatin bodies were filled with 50 mg powder Carbopol 934 P at the bottom on which a separating polymeric disc was placed. The polymeric mixture of ofloxacin (Table 1) was filled over it with light compression, and finally the capsule body was sealed with hard gelatin cap.  Figure 1 demonstrates the fabrication and drug release from prepared capsule.

### 2.8. *In Vitro* Buoyancy Studies

The *in vitro* buoyancy was determined by the floating time and was studied here by two methods. Firstly, by placing the formulated capsule in a beaker containing 100 mL of pH 1.2 HCl. The floating duration of all capsules was determined by visual observation (Figure 3). Secondly, the duration of buoyancy was determined in the USP dissolution Apparatus II in an acid environment. The time interval between the introduction of the capsule into the dissolution medium and its buoyancy to the top of dissolution medium was taken as the duration of buoyancy observed visually [17]. 

### 2.9. *In Vitro* Drug Release

The release of ofloxacin from the capsules was studied using USP type II (paddle) dissolution apparatus (EDT-08Lx, Electrolab, India). The dissolution media included 900 mL of phosphate buffer pH 1.2 (without pepsin) for entire study. The temperature was maintained at 37 ± 0.5°C with paddle rotation speed of 50 rpm. Five milliliters of aliquot were withdrawn at predetermined time intervals of 0.5, 1, 2, 3, 4, 5, 6, 7, 8, and 9 hours and filtered. The medium was replenished with 5 mL of fresh buffer each time. Sample was analyzed by using UV spectrophotometer (UV-1800 PC) at the wavelength of 291 nm. The studies were performed in triplicate.

### 2.10. Stability Study

To assess the drug and formulation stability, stability studies were carried out according to ICH guidelines [18]. The drug polymer mixtures were filled in hard gelatin capsule size 00 and stored in air tight glass container. Samples were placed in stability chamber (Remi Programmable Environmental Test Chamber, India) under accelerated storage conditions (45 ± 2°C, 75 ± 5% RH) for 3 months. At the end of studies, samples were evaluated for appearance, *in vitro* drug release, and infrared spectroscopy (Shimadzu FT-IR Affinity 1700). 

## 3. Results

### 3.1. Solubility of Ofloxacin at Different pH Range at Room Temperature

**Figure 2:**
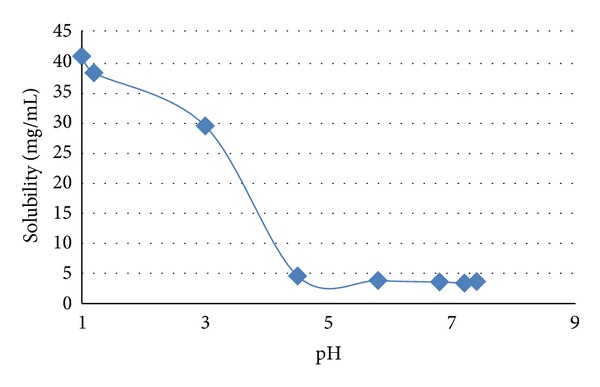
Graphical representation of ofloxacin solubility study curve at different pH range at room temperature.

**Figure 3:**
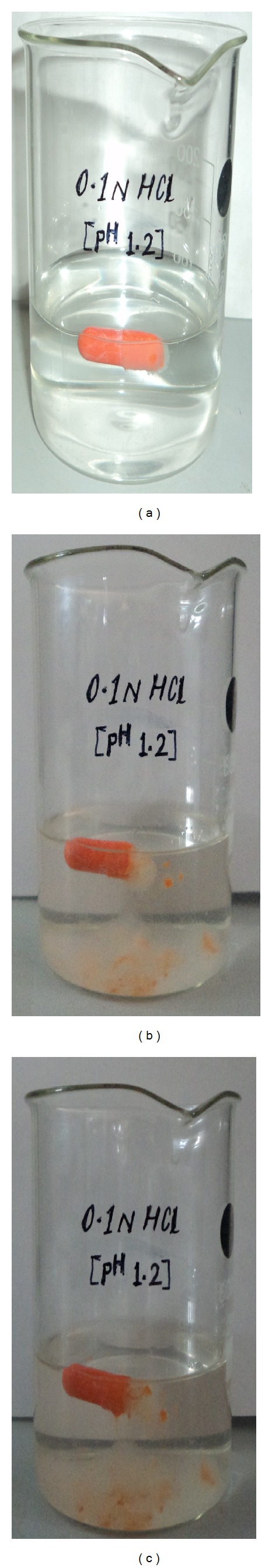
Floating behavior of NDCC in acidic buffer pH 1.2. The figure demonstrates the floating behavior of developed formulation F_E2_: (a) 0 hr, (b) 5 hrs, and (c) 9 hrs.

Solubility of Ofloxacin substantively decreased with an increase in a pH of media (Figure 2). It was found to be much higher in acidic pH compared to neutral and alkaline one. The solubility was found to be 41.2 mg/mL, 38.42 mg/mL, 29.57 mg/mL, 4.53 mg/mL, 3.79 mg/mL, 3.55 mg/mL, 3.39 mg/mL, and 3.24 mg/mL in media with pH 1, pH 1.2, pH 3, pH 4.5, pH 5.8, pH 6.8, pH 7.2, and pH 7.4, respectively.

### 3.2. Evaluation of Coating Thickness

The thickness measurement was performed to check uniform deposition of enteric coat on capsule shell body because it is necessary for the capsule to stay intact in acidic media for entire duration of residence in stomach. Initially, the bare capsule showed the thickness of 0.1104 ± 0.0016 mm, while after coating, the thickness appeared to be 0.1747 ± 0.0058 mm. Hence, the coating attained by the capsule shell was evaluated to be 0.0643 mm.

### 3.3. Buoyancy Time for the Various Formulations

Buoyancy of formulation depends on the type and concentration of excipients used. The formulations with HPMC grades showed better floating time while Carbopol 940 provided reduced floating property as negative effect on floating behavior of delivery system. This was previously suggested by Li et al. by moisture absorption isotherm of Carbopol 934P, HPMC K4M, and K100LV. The moisture gain for Carbopol 934P was significantly higher compared with HPMC K4M and K100LV (55% weight gain for Carbopol 934P versus ~33% for HPMC K4M and K100LV at RH of 95%). This results in a dramatic increase in the density of the GFDDS which, in turn, shows a corresponding decrease in the floating capacity of floating delivery devices. Xanthan gum depicted satisfactory floating property when used in higher concentration (60 mg). 

Table 3 reveals that floating property of formulation containing Carbopol 940 and Xanthan gum increases with an increase in polymer concentration. The formulations F_A1_, F_A2_, F_E1_, F_E2_, F_K1_, F_K2_, F_G1_, F_G2_, and F_X1_ showed desired floating time for more than 9 hrs and seemed to be developed formulations.

### 3.4. *In Vitro* Drug Release

**Figure 4:**
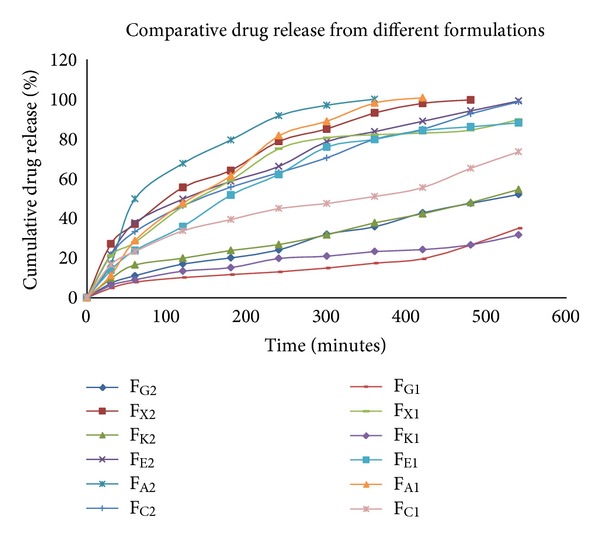
*In vitro* drug release of Ofloxacin using different polymers.

The sustained release polymers used in formulation on contact with aqueous media get hydrated forming gel matrix that entrapped the air responsible for buoyancy. This gel structure acts as a reservoir system for sustained drug release which is governed by slow diffusion through hydrated gel barrier [19, 20].  Figure 4 represents comparative release of ofloxacin from various formulations containing different sustained release polymers. The effective sustained release was obtained from all formulations. The formulations F_G1_, F_G2_, F_K1_, F_K2_, and F_C1_ showed much sustained drug release 35.01%, 52.01%, 31.66%, 54.52%, and 73.47%, respectively in 9 hours. The formulations F_E1_, F_E2_, F_X1_, and F_C2_ successfully sustained the drug release till 9 hours and depicted the drug release as 88.08%, 99.08%, 89.70%, and 98.65%, respectively. Formulations F_A1_, F_A2_, and F_X2_ showed the complete drug release within 6, 7, and 8 hours, respectively. The formulations F_E2_ and F_A2_ were able to sustain the drug release up to 9 hrs with considerably higher drug release 98.65% and 99.08% respectively, and proved to be developed batches. 

### 3.5. Release Kinetics

Release behavior of prepared formulations was essentially studied by various mathematical models. Equation (2) describes zero order kinetics, where the drug release rate in a system is independent of its concentration [21] and ideal to describe coated dosage forms or membrane controlled dosage form [22]. The first-order Equation (3) describes the release from system where release rate is concentration dependent [23, 24]; it described the release of drugs from insoluble matrix as a square root of time-dependent process based on Fickian diffusion equation (4). Korsmeyer-Peppas equation (5) suggests the drug release from swellable polymer [25, 26]. The Hixson-Crowell cube root law Equation (5) describes the release from systems where there is a change in surface area and diameter of particles or tablets.

(2) $$\begin{array}{c} Q_{t}=k_{0}t, \end{array}$$

(3) log⁡⁡Qt=log⁡⁡Q0−k1t,

(4) Qt=kHt1/2,

(5) MtM0=kKPtn,

(6) Q01/3−Qt1/3=kHCt,

where *Q*_*t*_ is the amount of drug released at time, *Q*_0_ is the initial amount of the drug in the formulation and *k*_0_, *k*_1_, *k*_H_, *k*_KP_, and *k*_HC_ are the release rate constants for zero order, first-order, Higuchi model, Korsmeyer-Peppas model and Hixson-Crowell model, respectively. In (6), *M*_*t*_ and *M*_0_ are the amount of drug released at time *t* and time 0 while *n* is the diffusional coefficient.

The graphs for each formulation plotted according to the above equations were used to calculate correlation factors (*r* ^2^) and release exponents (*n*). All formulations suggested the Korsmeyer-Peppas as best fit model except F_E2_ and F_X2_ where drug release was in concordance with Higuchi model. As the fabricated capsule is exposed only from single planar side where there is absence of edge effect on dissolution media, it would behave as polymeric film like geometry for drug delivery in diffusion study of different release exponent. The *n* value 0.5 < *n* < 1.0 indicates an anomalous drug transport. The release exponent of 0.5 can serve as an indication for diffusion controlled drug release [27, 28].

### 3.6. Stability Testing

Intention behind stability study of drug and polymer was to yield evidence regarding the quality of formulation which varies with time under the influence of various environmental factors such as temperature and humidity. The samples tested for drug release and infrared spectroscopy after the specified time proved that the formulation remained stable and absence of drug polymer interaction. The *in vitro* drug release from formulations subjected to stress condition was quite similar to that of initial formulations. Also, peaks observed in infrared spectroscopy of pure ofloxacin, *C* = 0 stretching vibration 1750–1700 cm^−1^and O–H stretching at 3050–3000 cm^−1^ remained unchanged in drug polymer mixture subjected to stability study indicating compatibility of ofloxacin with used polymers.

## 4. Discussion

### 4.1. Solubility Study of Ofloxacin at Different pH

The solubility of ofloxacin varied significantly with the change in pH of the media. In acidic media the solubility was found to be much higher compared to neutral and alkaline media. The solubility was highest (41.2 mg/mL) in pH 1 media which considerably decreased with increase in pH of solvent. The solubility drastically reduced to 29.4 mg/mL when the pH 3 media was used. Also, in neutral and alkaline media the solubility remained less than 4 mg/mL. The bioavailability of drug with higher dose gets affected by this pH dependent solubility condition.

### 4.2. Evaluation of Coating Thickness

The efficient thickness was achieved on capsule by Eudragit S100 coating. The thickness was sufficient to resist the deformation in acidic media for more than 2 hrs. From the results obtained by measurement of coating thickness, it can be assumed that there will not be any deformation of capsule body *in vivo* due to less deviation found in study; it indicates uniform deposition of enteric coat. It was also confirmed by visual observation of intactness of capsule shell in simulated gastric fluid (SGF) *in vitro* for more than 9 hrs.

### 4.3. *In Vitro* Buoyancy Studies

The formulations comprising of different grades of HPMC (F_A1_, F_A2_, F_E1_, F_E2_, F_K1_, and F_K2_) and Guar gum (F_G1_ and F_G2_) provided better floating property. These formulations showed floating time for more than 9 hrs. As Carbopol has negative effect on floating, formulations F_C1_ and F_C2_ failed to maintain the buoyancy for desired span. Formulations with Xanthan gum also exhibited the good buoyancy of capsules. However, the formulation F_X2_ failed to maintain buoyancy for 9 hrs where the concentration of Xanthan gum was lesser (40 mg).

### 4.4. *In Vitro* Drug Release

All the prepared formulations were able to control the release of the drug efficiently over the span of 9 hours, except the formulations F_A1_, F_A2_, and F_X2_ which showed total drug release within 7, 8, and 9 hours, respectively. Drug release was undesirably much sustained from formulations F_G1_, F_G2_, F_K1_, F_K2_, and F_C1_ which remained less than 50% till 9 hrs. The formulations comprising of HPMC polymers showed better results. However, F_A1_ and F_A2_ containing HPMC A failed to sustain the release up to 9 hrs. Formulations F_E2_ and F_C2_ produced the better results by providing maximum drug release in sustained manner till 9 hrs. Hence, it indicates that when HPMC E and Carbopol 940 were used in concentration of 40 mg, they exhibited desired outcome, as formulation F_C2_ has poor floating property which leads to unfavorable batch. Thus, F_E2_ turned out to be best developed formulation where 40 mg HPMC E15 was taken into account.

### 4.5. Drug Release Kinetics

All the formulations except F_E2_ and F_X2_ followed the Korsmeyer-Peppas model for drug release indicating the drug release from swellable polymers. The release exponent suggests the drug release mechanisms from polymeric controlled delivery systems. Korsmeyer-Peppas model was found to be best fitted with anomalous diffusion for all the formulations with *n* values between 0.5 and 1 except for formulation F_E2_ and F_X2_, where they followed Higuchi model and diffusion-controlled drug release mechanism. The developed formulation F_E2_ depicted the drug release according to Korsmeyer-Peppas model as shown in Table 4. 

### 4.6. Stability Studies

Stability study of formulations was investigated successfully indicating that formulation subjected to stress remained stable after 3 months. The drug release behavior remained similar to the unsubjected formulations. Also, FT-IR spectroscopy data reported no interaction between drug and polymers

## 5. Conclusion

The present study suggests that the use of NDCC for oral delivery of Ofloxacin could be an alternative to improve its systemic availability which could be regulated by the floating approach. As Ofloxacin efficiently absorbed from stomach, bioavailability of Ofloxacin could be considerably increased by gastroretentive NDCC. This study shows the release behavior and buoyancy of formulations by using various sustained release polymers. The release behavior of developed formulation F_E2_ showed release kinetics according to Korsmeyer-Peppas model indicating drug release from swellable polymer. Also, the floating behavior of formulations with Guar gum and HPMC grade polymers was satisfactory. The designed dosage system can have futuristic applications over payloads which require stomach-specific delivery. 

## Figures and Tables

**Table 1:** Salient features of GI tract.

Segment	Surface area	pH
Stomach	3.5 m^2^	1–3.5
Duodenum	2 m^2^	4–6.5
Jejunum	180 m^2^	5–7
Ileum	280 m^2^	6–8
Colon	1–3 m^2^	6–8

**Table 2:** Composition of formulations.

	Formulation codes											
Ingredients	F_A1_	F_A2_	F_E1_	F_E2_	F_K1_	F_K2_	F_C1_	F_C2_	F_G1_	F_G2_	F_X1_	F_X2_
	Quantities in mg											
Ofloxacin	200	200	200	200	200	200	200	200	200	200	200	200
HPMC A15	60	40										
HPMC E15			60	40								
HPMC K15					60	40						
Carbopol 940P							60	40				
Guar gum									60	40		
Xanthan gum											60	40
Carbopol 934P	50	50	50	50	50	50	50	50	50	50	50	50

**Table 3:** *In vitro* floating study of various formulations.

Formulation	Buoyancy time (Yang et al.)	Buoyancy time (Rosa et al.)
F_A1_	>9 hrs	>9 hrs
F_A2_	>9 hrs	>9 hrs
F_E1_	>9 hrs	>9 hrs
F_E2_	>9 hrs	>9 hrs
F_K1_	>9 hrs	>9 hrs
F_K2_	>9 hrs	>9 hrs
F_C1_	>4 hrs	>5 hrs
F_C2_	>4 hrs	>4 hrs
F_G1_	>9 hrs	>9 hrs
F_G2_	>9 hrs	>9 hrs
F_X1_	>9 hrs	>9 hrs
F_X2_	>7 hrs	>9 hrs

**Table 4:** Mathematical models for different formulations.

Formulation code	Correlation coefficient (*r* ^2^)					Release exponent (*n*)	Best fit model
	Zero order	First order	Higuchi	Korsmeyer-Peppas	Hixson-Crowell
F_A1_	0.947	0.924	0.975	0.993	0.690	0.8086	Korsmeyer-Peppas
F_A2_	0.857	0.973	0.965	0.977	0.587	0.6494	Korsmeyer-Peppas
F_E1_	0.917	0.988	0.979	0.994	0.642	0.6593	Korsmeyer-Peppas
F_E2_	0.909	0.875	0.995	0.971	0.566	0.4996	Higuchi
F_K1_	0.943	0.959	0.986	0.998	0.607	0.5318	Korsmeyer-Peppas
F_K2_	0.970	0.974	0.962	0.990	0.645	0.5511	Korsmeyer-Peppas
F_C1_	0.929	0.951	0.949	0.977	0.949	0.4786	Korsmeyer-Peppas
F_C2_	0.935	0.853	0.965	0.975	0.965	0.5089	Korsmeyer-Peppas
F_G1_	0.919	0.893	0.850	0.968	0.684	0.5764	Korsmeyer-Peppas
F_G2_	0.990	0.988	0.953	0.993	0.717	0.6751	Korsmeyer-Peppas
F_X1_	0.847	0.957	0.965	0.975	0.559	0.5291	Korsmeyer-Peppas
F_X2_	0.897	0.892	0.994	0.967	0.560	0.4870	Higuchi

## References

[1] Hwang S, Park H, Park K (1998). Gastric retentive drug-delivery systems. *Critical Reviews in Therapeutic Drug Carrier Systems*.

[2] Deshpande AA, Rhodes CT, Shah NH, Malick AW (1996). Controlled-release drug delivery systems for prolonged gastric residence: an overview. *Drug Development and Industrial Pharmacy*.

[3] Arora S, Ali J, Ahuja A, Khar RK, Baboota S (2005). Floating drug delivery systems: a review. *AAPS PharmSciTech*.

[4] Singh BN, Kim KH (2000). Floating drug delivery systems: an approach to oral controlled drug delivery via gastric retention. *Journal of Controlled Release*.

[5] Khosla R, Davis SS (1990). The effect of tablet size on the gastric emptying of non-disintegrating tablets. *International Journal of Pharmaceutics*.

[6] Davis S, Stockwell AF, Taylor MJ (1986). The effect of density on gastric emptying of single and multiple unit dosage forms. *Pharmaceutical Research*.

[7] Mojaverian P, Vlasses PH, Kellner PE, Rocci ML (1988). Effects of gender, posture, and age on gastric residence time of an indigestible solid: pharmaceutical considerations. *Pharmaceutical Research*.

[8] Rouge N, Allémann E, Gex-Fabry M (1998). Comparative pharmacokinetic study of a floating multiple-unit capsule, a high-density multiple-unit capsule and an immediate-release tablet containing 25 mg atenolol. *Pharmaceutica Acta Helvetiae*.

[9] Chen RN, Ho HO, Yu CY, Sheu MT (2010). Development of swelling/floating gastroretentive drug delivery system based on a combination of hydroxyethyl cellulose and sodium carboxymethyl cellulose for Losartan and its clinical relevance in healthy volunteers with CYP2C9 polymorphism. *European Journal of Pharmaceutical Sciences*.

[10] Liu Y, Zhang J, Gao Y, Zhu J (2011). Preparation and evaluation of glyceryl monooleate-coated hollow-bioadhesive microspheres for gastroretentive drug delivery. *International Journal of Pharmaceutics*.

[11] Gröning R, Berntgen M, Georgarakis M (1998). Acyclovir serum concentrations following peroral administration of magnetic depot tablets and the influence of extracorporal magnets to control gastrointestinal transit. *European Journal of Pharmaceutics and Biopharmaceutics*.

[12] Whitehead L, Fell JT, Collett JH, Sharma HL, Smith AM (1998). Floating dosage forms: an in vivo study demonstrating prolonged gastric retention. *Journal of Controlled Release*.

[13] Chavanpatil MD, Jain P, Chaudhari S, Shear R, Vavia PR (2006). Novel sustained release, swellable and bioadhesive gastroretentive drug delivery system for ofloxacin. *International Journal of Pharmaceutics*.

[14] Castro B, Gameiro P, Lima JLFC, Matos C, Reis S (2001). Interaction of drug with hexadecylphocholine micelles. Derivative spectroscopy, acid-base and solubility studies. *Materials Science and Engineering: C*.

[15] Mehta A (1986). Evaluation of fluid-bed process for enteric coating systems. *Pharmaceutical Technology*.

[16] Li S, Feld KM, Kowarski CR (1991). Preparation and evaluation of Eudragit acrylic resin for controlled drug release of pseudoephedrine hydrochloride. *Drug Development and Industrial Pharmacy*.

[17] Yang L, Eshraghi J, Fassihi R (1999). A new intragastric delivery system for the treatment of Helicobacter pylori associated gastric ulcer: in vitro evaluation. *Journal of Controlled Release*.

[18] Matthews BR (1999). Regulatory aspects of stability testing in Europe. *Drug Development and Industrial Pharmacy*.

[19] Huber HE, Dale LB, Christenson GL (1966). Utilization of hydrophilic gums for the control of drug release from tablet formulations. I. Disintegration and dissolution behavior. *Journal of Pharmaceutical Sciences*.

[20] Sheth PR, Tossounian J (1984). The hydrodynamically balanced system (HBS(TM)): a novel drug delivery system for oral use. *Drug Development and Industrial Pharmacy*.

[21] Hadjiioannou T, Christian GD, Koupparis MA, Macheras PE (1993). *Quantitative Calculations in Pharmaceutical Practice and Research*.

[22] Ocak F, Ağabeyoğlu I (1999). Development of a membrane-controlled transdermal therapeutic system containing isosorbide dinitrate. *International Journal of Pharmaceutics*.

[23] Bourne D (2002). *Modern Pharmaceutics*.

[24] Higuchi T (1963). Mechanism of sustained-action medication. Theoretical analysis of rate of release of solid drugs dispersed in solid matrices. *Journal of Pharmaceutical Sciences*.

[25] Korsmeyer RW, Lustig SR, Peppas NA (1986). Solute and penetrant diffusion in swellable polymers. I. Mathematical modeling. *Journal of Polymer Science: Polymer Physics Edition*.

[26] Korsmeyer RW, Von Meerwall E, Peppas NA (1986). Solute and penetrant diffusion in swellable polymers. II. Verification of theoretical models. *Journal of Polymer Science: Polymer Physics Edition*.

[27] Ritger PL, Peppas NA (1987). A simple equation for desciption of solute release I. Fickian and non-Fickian release from non-swellable devices in the form of slabs, spheres, cylinders or discs. *Journal of Controlled Release*.

[28] Ritger PL, Peppas NA (1987). A simple equation for description of solute release II. Fickian and anomalous release from swellable devices. *Journal of Controlled Release*.

